# Snake Venom L-Amino Acid Oxidases: Trends in Pharmacology and Biochemistry

**DOI:** 10.1155/2014/196754

**Published:** 2014-03-12

**Authors:** Luiz Fernando M. Izidoro, Juliana C. Sobrinho, Mirian M. Mendes, Tássia R. Costa, Amy N. Grabner, Veridiana M. Rodrigues, Saulo L. da Silva, Fernando B. Zanchi, Juliana P. Zuliani, Carla F. C. Fernandes, Leonardo A. Calderon, Rodrigo G. Stábeli, Andreimar M. Soares

**Affiliations:** ^1^Faculdade de Ciências Integradas do Pontal e Departamento de Genética e Bioquímica, Universidade Federal de Uberlândia (UFU), Uberlândia, MG, Brazil; ^2^Centro de Estudos de Biomoléculas Aplicadas à Saúde, (CEBio), Fundação Oswaldo Cruz, Fiocruz Rondônia e Departamento de Medicina, Universidade Federal de Rondônia (UNIR), Porto Velho, RO, Brazil; ^3^Departamento de Análises Clínicas, Toxicológicas e Bromatológicas, Faculdade de Ciências Farmacêuticas de Ribeirão Preto (FCFRP), Universidade de São Paulo (USP), Ribeirão Preto, SP, Brazil; ^4^Departamento de Química, Biotecnologia e Engenharia de Bioprocessos, Universidade Federal de São João del Rei (UFSJ), Campus Altoparaopeba, Ouro Branco, MG, Brazil

## Abstract

L-amino acid oxidases are enzymes found in several organisms, including venoms of snakes, where they contribute to the toxicity of ophidian envenomation. Their toxicity is primarily due to enzymatic activity, but other mechanisms have been proposed recently which require further investigation. L-amino acid oxidases exert biological and pharmacological effects, including actions on platelet aggregation and the induction of apoptosis, hemorrhage, and cytotoxicity. These proteins present a high biotechnological potential for the development of antimicrobial, antitumor, and antiprotozoan agents. This review provides an overview of the biochemical properties and pharmacological effects of snake venom L-amino acid oxidases, their structure/activity relationship, and supposed mechanisms of action described so far.

## 1. Composition of Snake Venoms

During the continuing evolution of snakes, according to Kardong [1], the development of more specialized glandular venom was essential in the emergence of biologically active substances capable of weakening prey to facilitate their capture. At first the discharge's main function was to lubricate the snake's food, but with the passage of time, some enzymes mixed with secretions allowing for the emergence of more elaborate and potentially toxic proteins, used in the immobilization of prey. A quantitative increase in the production of these secretions as well as a qualitative improvement of toxic proteins promoted a gain in absolute discretion in defense against predators [2]. Qualitatively, snake venoms consist of a mixture of protein with or without catalytic activity such as phospholipases A_2_ (PLA_2_), proteases, hyaluronidases, L-amino acid oxidases (LAAOs), acetylcholinesterases, growth factors, protein C activators, lectins, and von Willebrand factor-binding proteins; peptides mainly comprising bradykinin potentiators and disintegrins; low molecular weight organic compounds such as carbohydrates, serotonin, histamine, citrate, and nucleosides; and inorganic ions such as calcium, cobalt, magnesium, copper, iron, and potassium, as well as enzymatic inhibitors [3].

## 2. L-Amino Acid Oxidases

LAAOs are widely distributed in many different species including insects, fungi, bacteria, and snakes [4] and are even found in plants where one of their catalytic products, ammonia, is used as a nitrogen source in cell metabolism [5, 6].

LAAO activity was first observed by Krebs [7] in hepatic and renal tissue homogenates. Subsequently, Blanchard et al. [8] isolated the first LAAO from a rat kidney. Regarding snake venoms, this class of molecules was only detected in 1944 by Zeller and Maritiz [9] who studied the venom of* Vipera aspis*. In 1979, Iwanaga and Suzuki [10] described the potential of LAAOs as enzymes when observing a highly specific chemical reaction with L-amino acids. Snake venom LAAOs (SV-LAAOs) are usually homodimeric with cofactors FAD (Flavin Adenine Dinucleotide) or FMN (Flavin Mononucleotide) covalently linked to their chemical structure. The yellow color of venoms rich in these enzymes is related to the presence of the pigment riboflavin present in the cofactors, a fact that facilitates its purification. Quantitatively, there are inter- and intraspecific variations in the content of this enzyme in the whole venom (Table 1), and therefore there is color variance between the venoms. In exceptional cases, one gland of the same individual may produce yellow venom and the other gland colorless venom as observed in* Crotalus viridis helleri* [11].

In snake venoms, LAAOs are found in high concentrations that vary according to each species of snakse, which may contribute to the toxicity of the venom. LAAOs exhibit catalytic specificity for long chain hydrophobic and aromatic amino acids and are active in a wide range of pHs and temperatures. Their structures, molecular masses, and isoelectric points are quite varied. They are able to induce changes in platelet function, which cause local effects on plasma clotting disorders among other things. LAAOs are capable of inducing apoptosis in various cell lines and show antimicrobial and antiparasitic activity. According to Ande et al. [36] the existence of LAAOs may be a means of protection against natural agents, parasites, and bacteria.

## 3. Enzymatic Activity of L-Amino Acid Oxidases

**Figure 1:**
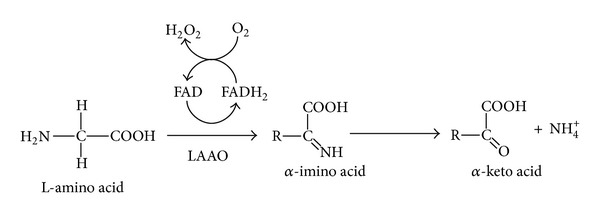
Mechanism of chemical reaction catalyzed by L-amino acid oxidases (LAAOs) [37].

LAAOs (EC 1.4.3.2) are flavoenzymes belonging to the class of oxidoreductases that catalyze the stereospecific oxidative deamination of L-amino acids. During the reduction half-reaction, the amino acid substrate is oxidized to an imino acid, with a concomitant reduction of the FAD cofactor. The imino acid then undergoes nonenzymatic hydrolysis, yielding *α*-keto acid and ammonia. Another oxidation half-reaction completes the catalytic cycle, reoxidizing FADH_2_ in the presence of molecular oxygen and thus generating hydrogen peroxide (Figure 1).

LAAOs are considered to be a class of multifunctional enzymes in view of their ability to produce hydrogen peroxide and ammonia, their participation in cell metabolism, and their possible protective effects, including their antiseptic and antimicrobial activities on different organisms. Furthermore, the correlation between the production of LAAOs and their utilization in metabolic pathways involving nitrogen, as well as the production of hydrogen peroxide, opens perspectives for new applications of these enzymes as bactericidal, antiviral, and antitumor agents, making them a promising biotechnological agent. Thus various research groups have studied LAAOs isolated from different snake species [12, 14, 15, 20, 21, 23, 24, 29, 30, 32–35, 38–48].

### 3.1. Importance of Hydrogen Peroxide

The hydrogen peroxide generated during the enzymatic reaction is a highly toxic oxygen reactive species that is capable of acting on nucleic acids, proteins, and plasma cell membranes [49]. This reactive oxygen species, according to Ande et al. [36], is formed extracellularly, may act directly on cell membranes by altering the permeability of the attacked area, and may also be involved in necrosis or apoptosis. The process of necrosis could be related to the direct action of hydrogen peroxide on the plasma cell membrane, since within the mechanism of apoptosis the development of morphological, biochemical, and molecular changes leads to cell death. The most common morphological changes were chromatin condensation, reduction and disintegration of nucleolus volume, and others. It also seems to be involved in the cytotoxic mechanisms of the enzyme which may ultimately represent another defense mechanism of the organism in response to the environment.

### 3.2. Enzymatic Kinetics of L-Amino Acid Oxidases

Kinetic studies suggest that LAAOs present preferential catalytic specificity for hydrophobic and aromatic L-amino acids (Table 1), whereas their affinity for polar and basic amino acids is low [12, 15, 16, 21, 24, 28, 30, 32, 33, 35, 44, 46, 47]. Positively charged amino acids such as L-lysine and L-arginine present unfavorable electrostatic interactions with the catalytic site of the enzyme [50].

Oxidation catalyzed by LAAOs follows Michaelis-Menten kinetics [6, 14, 15, 28, 29, 31, 33, 34, 46, 48, 49, 51–54]. The kinetic parameters *K*_*m*_ and* K*_cat_ ****shown in Table 2 are very useful for the study and comparison of different enzymes in relation to their substrate. Each enzyme presents optimal *K*_*m*_ and* K*_cat_ values that reflect the cellular environment, substrate concentration, and chemical characteristics of the reaction catalyzed. *K*_*m*_, the Michaelis-Menten constant, often used as an indicator of the affinity of the enzyme for the substrate, is specific for each L-amino acid oxidized by LAAOs [29], whereas* K*_cat_ is the number of substrate molecules converted into the product per unit of time. The maximum velocity (*V*_max⁡_) reached in each enzymatic reaction is associated with the concentration of the substrate present in the medium and with *K*_*m*_ and is also specific for each substrate [12, 15, 16, 21, 31, 48, 49, 54].

#### 3.2.1. Effect of pH on the Enzymatic Kinetics of L-Amino Acid Oxidases

The oxidation of L-amino acids by LAAOs happens in a wide range of pHs. This maximal specific activity of each LAAO is related to the optimum pH for each type of amino acid acting as the substrate [58]. Paik and Kim [51] extensively studied the relationship between pH and substrate reactivity for LAAOs from snake venom and found different pH curves depending on the amino acid used as the substrate. Solis et al. [59] studied the action of LAAOs isolated from the venom of* Bothrops brazili* on the substrates L-leucine, L-methionine, L-phenylalanine, and L-arginine and observed that the enzyme remained active in a wide range of pH values; however the activity was highest at a pH of 8.5. Other amino acids such as L-isoleucine, L-tryptophan, and L-lysine showed optimum pHs of 7.5, 8.0 and 9.0, respectively; indeed various LAAOs also catalyze specific oxidoreduction reactions within a broad range of medium pHs [6, 15, 21, 29, 31, 34, 44, 47, 53]. The different profiles of specificity in terms of substrate and pH are related to the acid-base behavior of the enzyme in response to the amino acid. At a certain pH, both the enzyme and the substrate are in ionic equilibrium, permitting a better fit of the substrate in the active site of the enzyme and consequent maximum oxidation.

Snake venom LAAOs can suffer two types of reversible inactivation. One factor inducing inactivation is a change in pH to values close to neutral, resulting in a spontaneous structural change of the enzyme to its inactive configuration. If the pH is lowered, the active conformation of the enzyme is restored. The steady state is reached at a pH ranging from 5.5 to 7.5, and inactivation is more extensive at more alkaline pH levels [60]. This type of inactivation can be prevented by the addition of monovalent anions, substrates, and substrate analogs and is characterized by high activation energy.

#### 3.2.2. Effect of Metal Ions and Enzymatic Inhibitors on the Enzymatic Kinetics of L-Amino Acid Oxidases

Mackessy [61] fractionated the venom of* Crotalus ruber ruber* obtaining proteases, phosphodiesterases, and LAAOs. The activity of these enzymes, including that of the LAAOs, was inhibited in the presence of EDTA, N-ethylmaleimide, and 1,10-phenanthroline, as well as PMSF and glutathione. In the presence of enzymatic inhibitors, as mentioned above, LAAO cofactors NAD or FAD are reduced, causing inactivation of the enzyme [62].

Different bivalent ions can activate or inhibit the specific activity of some LAAOs. The LAAO of* Crotalus adamanteus* requires Mg^2+^ [51], whereas the enzymes of* Lachesis muta* and* Bothrops brazili* [59, 63] are inhibited in the presence of Zn^2+^. Other ions such as manganese and calcium do not affect the activity of these enzymes. The inhibitory action of these ions might be related to their ability to reversibly bind to thiol groups of cysteines present in the active site of the enzyme, reducing its activity [64], so many pharmacological activities of sv-LAAOs are compromised in the presence of some specific ions.

#### 3.2.3. Effect of Temperature on the Enzymatic Activity of L-Amino Acid Oxidases

The specific activity of some LAAOs depends on the experimental temperature. These enzymes remain active for a variable period of time at a broad range of temperatures (0° to close to 50°C) [21, 24, 28, 30, 32–34, 44, 53, 59]. Exposure to temperatures higher than 55°C results in a gradual decrease in activity caused by disruptions in hydrophobic interactions and hydrogen bonds between the different subunits of the enzyme. Temperatures lower than 25°C are associated with increased inactivation of the enzyme [24, 28, 30, 33, 34, 59, 65]. Moreover, LAAOs are also progressively inactivated when submitted to freezing or lyophilization [15, 24, 28, 30, 33, 66]. These types of inactivation by freezing, and also by alterations in pH as cited above, induce substantial conformational changes that can be demonstrated by circular dichroism [37]. These changes involve alterations in the binding of the enzyme to substrates and lack of binding to arachidonic acid (competitive inhibitor), as well as alterations in the affinity of the flavin coenzyme for electrons. Reversible inactivation by freezing involves specific regions of the catalytic site of the enzyme, affecting the redox properties of the cofactor-substrate complex [60, 67] and decreasing catalytic activity.

## 4. Purification of L-Amino Acid Oxidases

The first reports of isolation of LAAOs date back to the 1950s when Singer and Kearney [65] characterized an LAAO from* Agkistrodon piscivorus* snake venom. Later Wellner and Meister [68] obtained the crystal structure of LAAO purified from* Crotalus adamanteus* venom.

Snake venom LAAOs have been purified by fast and efficient chromatographic processes, including by size exclusion, ion-exchange, hydrophobic interaction, and affinity chromatographies (Table 1). A large number of these proteins have been isolated using basically the same chromatographic strategy, that is, fractionation of the venom by size exclusion chromatography, followed by hydrophobic interaction chromatography of the fractions of interest. This step can be repeated and, finally, the highly purified protein is applied to reverse-phase HPLC. However, each research group has adapted the steps of isolation to its specific protein and laboratory conditions. Numerous LAAOs have been isolated from different species:* Trimeresurus mucrosquamatus* [52, 69],* Trimeresurus jerdonii* [70],* Agkistrodon halys pallas* [71],* Agkistrodon halys blomhoffii* [72],* Ophiophagus hannah* [57],* Lachesis muta muta* [73],* Naja naja kaouthia* [56], and* Calloselasma rhodostoma* [53, 74]. Various other LAAOs were also purified following the same steps, indicating the process efficiency (Table 1).

## 5. Biochemical Characterization of L-Amino Acid Oxidases

When analyzed under nondenaturing conditions, LAAOs are usually noncovalently linked homodimeric proteins with a molecular mass of approximately 110–150 kDa. Examples include the LAAO of* Agkistrodon contortrix laticinctus* [75], LAAO of* Trimeresurus mucrosquamatus* [69], Balt-LAAO-I of* Bothrops alternatus* [35], CascaLAO of* Crotalus durissus cascavella* [31], BpirLAAO-I of* Bothrops pirajai* [32], LAAO of* Vipera berus berus* [33], LAAO of* Vipera lebetina* [30], Akbu-LAAO of* Agkistrodon blomhoffii ussurensis* [19], BmooLAAO-I of* Bothrops moojeni* [66], LAAO of* Naja naja oxiana* [28], SSAP of* Sebastes schlegeli* [76], Bp-LAAO of* Bothrops pauloensis* [24], DRS-LAAO of* Daboia russelii siamensis* [47], Akbu-LAAO of* Agkistrodon blomhoffii ussurensis* [19], BmarLAAO of* Bothrops marajoensis* [20], LAO* Bungarus caeruleus* [21], LmLAAO of* Lachesis muta* [15], and DrLAO of* Daboia russelii* [16]. When these toxins are treated under denaturing conditions, the molecular mass of each monomer determined by mass spectrometry is about 50–70 kDa (Table 1).

This variation in molecular mass among different LAAOs might be related to the sites of glycosylation since these enzymes are considered to be glycoproteins [14, 24, 28, 30, 32–35, 66, 69, 77, 78]. The association of carbohydrates with the structure of LAAOs was first detected by the method described by Lowry et al. [79]. This class of enzymes is characterized by a variable percentage of sugars which vary according to snake species: 4% in* Calloselasma rhodostoma*, 2.64% in* Bothrops brazili*, 3.6% in* Bothrops jararaca*, 2 to 5% in* Crotalus adamanteus*, 15% in* Bothrops alternatus*, 13–16% in* Bothrops moojeni*, 12% in* Bothrops atrox,* and, 25% in* Bungarus caeruleus* [21, 25, 35, 53, 59, 66, 80, 81], respectively. Some carbohydrates such as fucose, mannose, galactose, N-Acetylglucosamine, and sialic acid have been identified as associated with these enzymes, accounting for approximately 5.4% (w/w) of total proteins [59, 82, 83]. These sugars are linked to the enzyme through N-glycosidic bonds and probably modulate its physicochemical properties, increasing the solubility and viscosity of the protein and maintaining the stability of electrical charges [34, 66]. Studies have demonstrated that some LAAOs do not lose their catalytic activity after deglycosylation assays using O-glycosidase and PNGase F [24, 27, 32, 35, 66, 78], a finding suggesting that the carbohydrate moiety of the enzyme only plays a structural role or protects the enzyme against proteolysis since snake venoms are rich in proteolytic enzymes [84].

Most LAAOs described so far are variably acidic, with isoelectric points above 4.4 (Table 1). In contrast, some LAAOs are slightly basic and present an isoelectric point of 8.0 or higher, including the LAAO of* Trimeresurus flavoviridis* with a pI of 8.4 [29], LAAO of* Naja naja kaouthia* with a pI of 8.1 [85], LAAO of* Agkistrodon acutus* with a pI of 8.2 [86], and LAAO of* N. naja oxiana* with a pI > 8 [28]. Isoforms of the same LAAO are often present in the same venom, which can be acidic, neutral, or basic [87]. This difference in charge density may alter the pharmacological activities of LAAOs as observed with other snake venom enzymes.

## 6. Antigenicity of L-Amino Acid Oxidases

In general, snake venoms are strong antigenic inductors due to their high protein content. The variability in snake venom composition raises an additional problem for the production of antivenom serum and thus provides a commercial incentive for the manufacturers of therapeutic agents against ophidian envenomation. Particularly, inter- and intraspecific variations in snake venom composition have been demonstrated to affect the neutralization capacity of antivenom sera [88].

Various studies have been carried out to develop alternative methods to improve neutralization of the toxic effects of snake venom envenomation. Our knowledge about immunological cross-reactivity of venoms has evolved from experimental evidence obtained using different approaches. The phenomenon of cross-reactivity between snake venoms is related to the observation that antiserum specifically prepared against the venom of one type of snake may react with other snake venoms [89]. Studies on the cross-reactivity of snake venoms suggest the apparent lack of a correlation between cross-reactions and phylogeny, implying that the results obtained based on antigen recognition do not completely reflect the molecular evolution of snake venoms [90, 91]. The specificity of snake venom antibodies against a fragment of* Bothrops moojeni* LAAO shows that cross-reactivity is mediated, at least in part, by antibodies that are able to recognize another functional protein [92]. This difficulty in neutralizing venoms is mainly related to the damage at the site of the bite.

## 7. Structural and Molecular Characteristics of L-Amino Acid Oxidases

The development of recombinant DNA techniques and nucleotide and amino acid sequencing has permitted the creation of databases that are shared by various researchers in order to identify the composition of each venom and the key activities of each protein. The N-terminal amino acid sequences of various LAAOs from the snake families, Viperidae and Elapidae, were deduced by Edman degradation, and alignment of these sequences always showed a high identity, even when toxins originating from distinct snake species were compared [31, 32, 35, 75, 83, 93, 94]. cDNA analysis using yeast or* Escherichia coli* as expression vectors showed that partial sequences of venom LAAOs from different snake species also present highly conserved regions along the primary structure of the protein, characterizing high identity between these enzymes [14, 24, 27, 34, 66, 72, 75, 95]. The number of base pairs of these sequenced toxins is highly variable, and most sequences are deposited in the NCBI database (Table 3).

Macheroux et al. [37] deduced the complete sequence of* Calloselasma rhosdostoma* LAAO from cDNA, with the sequence showing a high identity with LAAOs from* Crotalus adamanteus* and* Crotalus atrox*.

cDNA sequencing of LAAOs provides important information for the structural understanding of this class of still poorly explored enzymes. Ali et al. [83] demonstrated the presence of a highly conserved *βαβ*-fold domain in the N-terminal region of an LAAO from* Eristicophis macmahoni* venom, which is responsible for binding the FAD cofactor. Zhang et al. [86] identified a change to asparagine in the second amino acid residue of the N-terminal region of AHP-LAAO from* A. halys pallas* venom, which might play an important role in enzymatic activity since this region is involved in many effects induced by the enzyme.

Multiple alignment of the primary structure of a* Calloselasma rhodostoma* LAAO showed a high similarity (>84%) with other snake venom LAAOs (Table 4). Phylogenetic comparisons between FAD-dependent snake venom LAAOs and other FAD-dependent oxidases, such as monoaminoxidase (MAO), D-amino acid oxidase, and tryptophan 2-monooxygenase, reveal only distant relationships. However, all LAAOs share a highly conserved dinucleotide-binding region with MAO, D-amino acid oxidase, tryptophan 2-monooxygenase, and various other proteins that may also require FAD [37].

**Figure 2:**
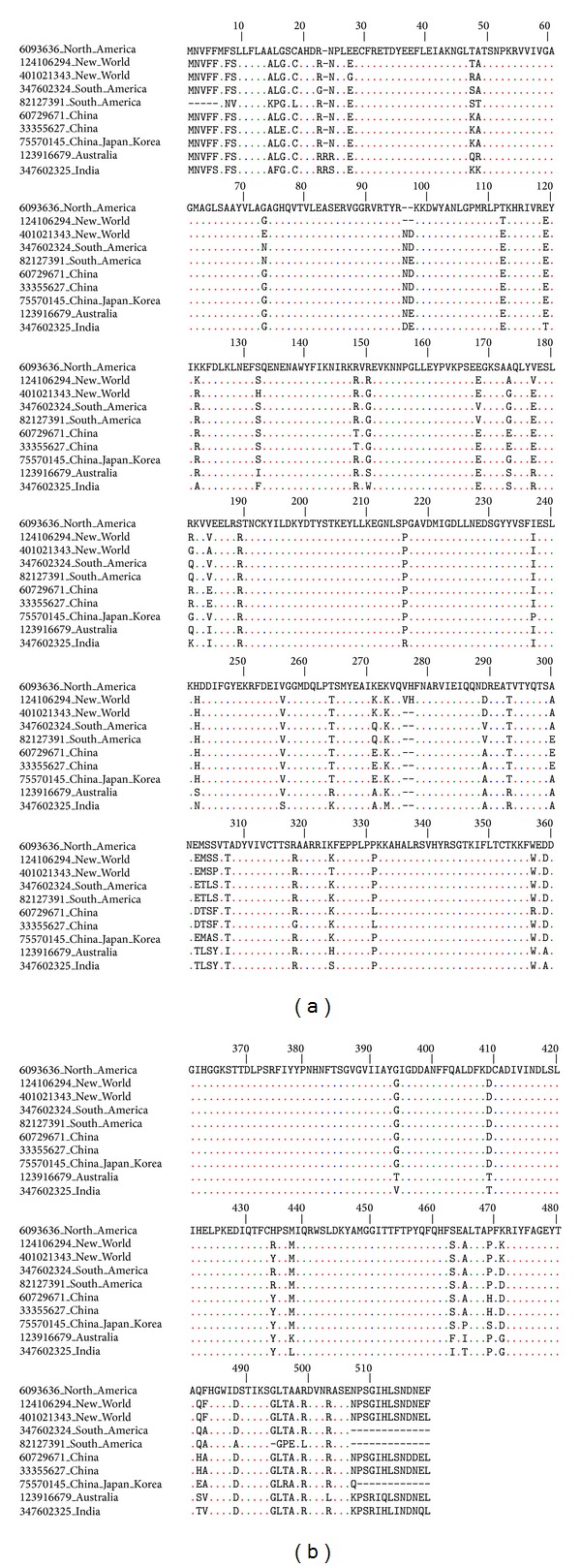
Sequence alignment of L-amino acid oxidases from snake venoms of some regions of the world. The alignment was performed using the program ClustalW [101]. Only nonconserved amino acids are showed.

Sequences of LAAOs were aligned and analyzed according to the region of the world. The results of the alignment are shown in order of alignment (Figure 2). Most dissimilar regions were found in the C- and N-termini, and higher conservation between sequences was seen in the territories that were occupied more recently by humans (North America and South America). The percentage of global alignment resulted in ~60% similarity.

**Figure 3:**
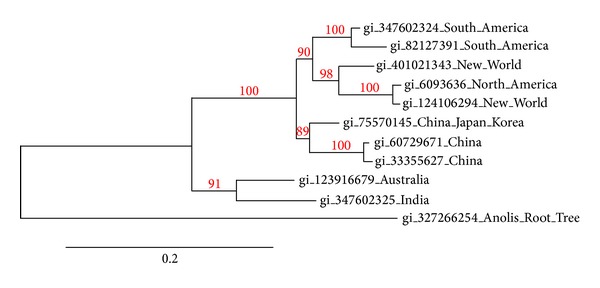
Phylogenetic representation of amino acid sequence alignments of L-amino acid oxidases from snake venoms of some regions of the world. Trees were obtained as described in the Methods section at http://www.phylogeny.fr/. Numbers close to the nodes represent the support value for each branch. The region of the world is shown after the accession numbers. The sequence gi*|*327266254_Anolis_Root_Tree is the root control of the tree.

In the phylogenetic tree (Figure 3), four groups may be distinguished: first South America; second North America and the New World; third China, Japan, and Korea; and fourth Australia and India. The sequence gi*|*327266254_Anolis_Root_Tree is an LAAO of the lizard* Anolis carolinensis* that was included for the control protocol. Phylogenetic analyses were run using the website http://www.phylogeny.fr/ [96]. Sequences were aligned using the MUSCLE program [97] according to the mer distances clustered by UGPMA. The Gblocks program [98] was used to eliminate poorly aligned positions and divergent regions. PhyML 3.0 was used for phylogenies [99] including substitution models WAG for proteins. The ALTR test (SH-like) was used to access the support values of each branch [100].

Generally, the composition of LAAOs is quantitatively similar, with many asparagine, glutamic acid, and aspartic acid residues and few methionine and tryptophan residues. The number of cysteine residues varies, indicating variations in the tertiary structure of these enzymes [35, 83, 102].

The cDNA-deduced sequence of various snake LAAOs is characterized by the presence of a highly conserved *βαβ* domain in the N-terminal region that is rich in glutamic acid residues and possibly functions as a binding site for the FAD cofactor, which is fundamental for the generation of hydrogen peroxide [37, 94]. Indeed, determination of the N-terminal region of LNV-LAAO isolated from* Eristicophis macmahoni* venom by Ali et al. [103] showed similarity with other snake venom LAAOs in terms of the large number of glutamic acid residues found in this region, suggesting an important functional role of the N-terminal region of these enzymes [31, 35].

**Figure 4:**
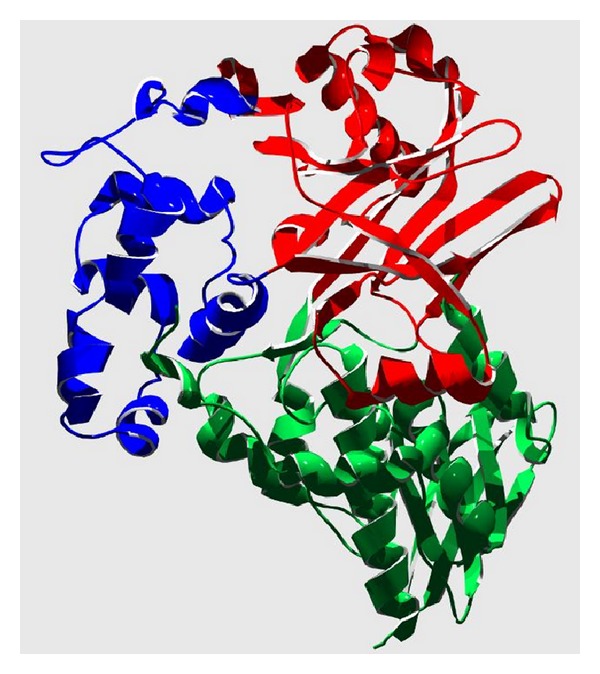
Structure of LAAO from* Calloselasma rhodostoma* (PDB code 2IID) with a resolution of 1.80 Å [50]. The structure was shown in ribbon representation using Swiss-PDBViewer software with renderization POV-ray [104]. In blue is the *α*-helical domain, in red is the substrate-binding domain, and in green is the FAD-binding domain.

The structure of LAAO from* Calloselasma rhodostoma* was determined in the presence of the ligands citrate, aminobenzoate, and phenylalanine. This analysis showed that the protein consists of three domains: an FAD-binding domain, a substrate-binding domain, and an *α*-helical domain (Figure 4). The interface between the *α*-helical domain and the substrate-binding domain forms a 25 Å long funnel, which provides access to the active site. Three aminobenzoate molecules are visible along the funnel, a finding suggesting the trajectory of the substrate to the active site.

The innermost aminobenzoate molecule forms a hydrogen bond with the active site residues, Arg90 and Gly464, and the aromatic portion of the ligand is located in a hydrophobic region. These interactions mimic binding of natural substrates.

Analysis of the surface of the* Calloselasma rhodostoma* LAAO active site showed that the recess has a long Y-shape which allows the substrate to interact with the enzyme in such a way that one portion of the input channel interacts with O_2_, and the other is where product release occurs [50]. According to these authors, the active site is dynamic and can undergo conformational changes due to the presence of two amino acid residues, His223 and Arg322. Both residues are located along the driveway to the substrate to the active center. These amino acids can take on two different conformations (A and B), and the His_223_ spends 40% of its time as conformation A and 60% as B. The His_223_ conformation A has an imidazole side chain group which is stabilized with the aid of a water molecule through hydrogen bonding with its neighbors Glu_209_ and Ser_220_. In conformation B, the imidazole ring is attached to a neighboring phenyl group at a distance of 3.1 Å. The lack of hydrogen bonding with neighboring side chains increases the mobility of this residue. The movements of the imidazole ring of the side chain are probably related to the mechanism of deprotonation of the substrate. Minor conformational changes are observed for Arg_322_. The two types of conformation of arginine differ in the position of carbons C*δ* and C*γ* depending on angles between −84° and 73° and the conformation to B.

The position of the guanidine group of the side chain is similar in both conformations, with the N*ε* stabilized through hydrogen bonding with the hydroxyl of Thr_432_. The guanidine group may also interact with the side chains of amino acids Glu_209_ and Glu_219_, making them stable. The movements observed in the side chain of His_223_ and Arg_322_ may be associated with the attachment and release of the substrate and product, respectively.

According to Moustafa et al. [50], during the binding of the substrate to the catalytic site of the enzyme, His_223_ assumes conformation A, blocking the entry of oxygen, thus allowing for the entry of the substrate. When the chemical reaction happens, His_223_ assumes conformation B again, unlocking the entry of oxygen to release the product. The movements in the side chain of Arg_322_, which change the conformation from form A to form B, are favorable hydrophobic interactions occurring between the aliphatic amino acid side chain and the aromatic ring of the enzyme substrate. After the enzymatic reaction occurs, it returns to form B to facilitate the release of the product. The interaction of the substrate with the active site of the enzyme is mediated by the guanidine group of Arg90 and hydrogen bonding with the hydroxyl of Tyr_372_. The amino group of the substrate forms hydrogen bonds with the carbonyl oxygen atom of the Gly_464_ residue, and then the side chains of the ligands form hydrophobic interactions with the side chains of Ile_430_ and Ile_374_ and Phe_227_. One of the two oxygen atoms of the carboxylic group of the substrate becomes involved with a water molecule by hydrogen bonding with the flavin N-5, as well as the amino group of the side chain of Lys_326_. Thus, the *α* carbon undergoes oxidative attack, generating the product.

Comparisons between the structure of snake venom LAAOs and mammalian D-amino acid oxidases reveal significant differences in the way the substrate arrives at the active site. In addition, a mirror-symmetrical relationship between the two substrate binding sites is observed, which facilitates enantiomer selectivity while the arrangement of atoms involved in catalysis is preserved [102].

## 8. Biological Effects of L-Amino Acid Oxidases

Since the 1950s when the isolation of different snake venom LAAOs from different species was initiated, studies to identify their activities and to understand their mode of action have also increased. As a result, different assays for the characterization of the toxic and pharmacological effects of these enzymes were standardized to obtain a better understanding. Until the 1990s, studies were restricted to the investigation of the structural and functional characteristics of these enzymes [4], whereas today* in vitro* and* in vivo* studies allow for a broader characterization and understanding of their effects and medical and pharmacological importance.

A range of researchers have characterized the functional properties of crude venoms from different snakes and observed that many of their toxic and pharmacological activities can be attributed to LAAOs [11, 100, 102, 105]. Comparative studies analyzing different snake venoms have demonstrated distinct effects induced by venoms that contain LAAO to a greater or lesser extent [106, 107]. Many of these effects seem to be related, at least in part, to hydrogen peroxide, a secondary product formed during the chemical reaction catalyzed by LAAOs. Du and Clemetson [4] reported a lack of evidence explaining such data, but different studies confirm that the addition of catalase in the presence of LAAOs completely suppresses the toxic effects of these enzymes [14, 20, 21, 24, 25, 27, 28, 30–34, 47–49, 66, 76–78, 95].

### 8.1. Local Alterations Induced by L-Amino Acid Oxidases

#### 8.1.1. Hemorrhage

The classical route of induction of hemorrhagic processes by snake venoms involves the degradation of extracellular matrix proteins of vascular endothelium. Souza et al. [75] proposed that snake venom LAAOs trigger a process of apoptosis in vascular endothelial cells, causing rupture of the endothelium and concomitant leakage of blood to the interstice. A few LAAOs isolated from snake venoms are able to induce hemorrhage, including ACL-LAO from* Agkistrodon contortrix laticinctus* [75], Balt-LAAO-I from* Bothrops alternatus* [35], ABU-LAO from* Agkistrodon blomhoffii ussurensis* [29], and BatroxLAAO from* Bothrops atrox* [25].

#### 8.1.2. Edema

The edematogenic activity of snake venoms is explained by an increase in vascular permeability that results in the leakage of fluid from blood vessels to the interstitial space of tissues. Some LAAOs have been described as edematogenic, including LNV-LAAO from* Eristicophis macmahoni* [83], TM-LAO from* Trimeresurus mucrosquamatus* [108], Balt-LAAO-I from* Bothrops alternatus* [35], BpirLAAO-I from* Bothrops pirajai* [32], ABU-LAO from* Agkistrodon blomhoffii ussurensis* [29], BmooLAAO-I from* Bothrops moojeni* [66], BatroxLAAO from* Bothrops atrox* [25], BF-LAAO from* Bungarus fasciatus* [55], and DrLAO from* Daboia russelii* [16].

Few studies have been conducted to determine the true mode of action of LAAOs in the induction of edema compared to other classes of snake toxins. Studies investigating different snake toxins revealed that the action of these compounds is related to the stimulation and release of inflammatory mediators such as histamine, prostaglandin, kinins, and serotonin [103]. On the other hand, the edematogenic activity of LAAOs does not seem to be mediated by the mechanisms described for other toxins since these enzymes do not lose their activity in the presence of antihistamines. Indeed, Ali et al. [103] observed that the edematogenic activity of* Ophiophagus hannah* LAAO was not inhibited in the presence of dexamethasone. However, the activity of this enzyme was completely suppressed when treated with glutathione, indicating that edema induced by LAAO is directly related to the presence of hydrogen peroxide.

### 8.2. Systemic Alterations Induced by L-Amino Acid Oxidases 

#### 8.2.1. Platelet Aggregation

The activity of LAAOs on platelets is still controversial, with a variable potential of these enzymes to inhibit or induce platelet aggregation. LAAOs isolated from the venoms of* Echis coloratus* [109],* A. h. blomhoffii* [72],* Vipera berus berus* [33],* Vipera lebetina* [30],* Bothrops leucurus* [17],* Ophiophagus hannah* [14],* Agkistrodon blomhoffii ussurensis* [29],* Naja naja oxiana* [28], and* Daboia russelii siamensis* [47] present inhibitory activity on platelet aggregation. Du and Clemetson [4] believe that hydrogen peroxide released by enzymatic action may interfere with the interaction between platelet receptors (GPIIb/IIIa) and fibrinogen, thus impairing the mechanism of aggregation. According to Zhong et al. [47], hydrogen peroxide produced during the catalytic reaction plays a fundamental role in the inhibition of platelet function, but the exact mechanism is still unclear. On the other hand, studies have shown the ability of various LAAOs to induce platelet aggregation [21, 24, 25, 31, 32, 35, 46, 69, 70]. However, this effect was always suppressed in the presence of catalase, indomethacin, and/or aspirin. Thus, the authors suggested that hydrogen peroxide production during oxidation of the substrate is related to this activity. In addition, the possible inhibition of endogenous PLA_2_ may play an important role in platelet aggregation. According to Sakurai et al. [85], the controversial results reported might be associated with differences in experimental procedures or plasma preparation.

Nathan et al. [109] demonstrated that the LAAO isolated from* O. hannah* venom induces platelet aggregation. The authors proposed that this effect may not be dependent on ADP but requires thromboxane A_2_ since they observed no change in aggregation activity when the creatine phosphokinase/creatine phosphate system, which consumes ADP, was added. In contrast, platelet aggregation was inhibited when aspirin and indomethacin were added in the presence of this LAAO. Catalase and EDTA also inhibited the activity of the enzyme. The authors therefore suggested that the induction of platelet aggregation by this enzyme is intimately related to the formation of hydrogen peroxide and the subsequent synthesis of thromboxane A_2_ which requires Ca^2+^, independent of the release of ADP. According to Zhong et al. [47], the role of hydrogen peroxide in the process of inducting platelet aggregation remains uncertain since recent studies indicate that it is unlikely that hydrogen peroxide alone is responsible for the biological activities of LAAOs; that is, other mechanisms are probably triggered and cause a potent biological response.

#### 8.2.2. Effects on Blood Coagulation

Alterations in blood coagulation induced by snake venoms have also been the target of many studies conducted in Brazil since the 1960s. This is not only due to the large amount of snake bites, but also because these venoms have become tools for the study of the complex processes involved in blood coagulation. These venoms are also used as auxiliary tools for clinical diagnosis and as therapeutic agents to provoke defibrinogenation during thrombosis treatment. Li et al. [110] isolated an LAAO from* Agkistrodon halys blomhoffii* venom, referred to as M-LAO, which presented high anticoagulant potential. This enzyme was found to inhibit the activity of factor IX, involved in the process of coagulation, in a time- and dose-dependent manner. M-LAO showed the same anticoagulant potential when membrane phospholipids were added, thus demonstrating that its inhibitory activity is directly related to the depletion of factor IX activity, destabilizing the intrinsic pathway of blood coagulation.

## 9. Pharmacological Effects of L-Amino Acid Oxidases

### 9.1. Antiviral Effect of L-Amino Acid Oxidases

The antiviral effect of LAAOs has yet to be well explored. One study reports the possible inhibition of HIV-1 replication by an LAAO isolated from* Trimeresurus stejnegeri* venom, called TSV-LAO [112]. This activity was demonstrated by a reduction in protein p24 production, which indicates HIV-1 replication and a decrease in syncytium formation. TSV-LAO was unable to block the fusion between HIV-1 and host cells, suggesting that this enzyme does not interfere with the absorption and/or binding of the virus to the host cell.

The antiviral potential of LAAOs was also studied by Sant'Ana et al. [27], who treated cells infected with DENV-3 virus strains, the etiological agent of dengue, with BjarLAAO-I isolated from* Bothrops jararaca* snake venom. The treatment's efficiency was demonstrated by a reduction of viral load in previously infected C6/36 cells exposed to the toxin when compared to controls of unexposed infected cells.

### 9.2. Antiparasitic Effect of L-Amino Acid Oxidases

Inhibitory activities on the growth of* Trypanosoma cruzi* and* Leishmania donovani infantum* or other species of* Leishmania* have been reported for different snake venoms [113]. This antiparasitic effect might be attributed to the activity of LAAOs [4, 15, 17, 20, 24, 27, 31, 32, 66, 78, 83, 95, 114].

Leishmaniasis is caused by parasites of the genus* Leishmania* spp. and is transmitted to the vertebrate host through the bite of mosquitoes of the genera* Lutzomyia* (Old World) and* Phlebotomus* (New World). Clinical manifestations observed in patients vary according to parasite species, with these parasites being widely distributed in tropical and subtropical regions worldwide. Leishmaniasis comprises a broad spectrum of infectious complications ranging from skin ulcerations to progressive and lethal visceral infections [115]. The first-line drugs for the treatment of this disease are pentavalent antimonials, which have serious side effects and to which the target parasites have shown clinical resistance. Thus, the study of new antiparasitic compounds from different sources, including those with antileishmanial activity, is of biotechnological and medical interest.

Chagas disease is caused by the parasite* Trypanosoma cruzi* and is transmitted by the vector* Triatoma infestans* (“barbeiro”). Epimastigotes are the potentially infectious form. The most common clinical manifestation observed during the chronic phase is cardiomyopathy, which often leads to death of the patient.

The leishmanicidal/trypanocidal effect of LAAOs purified from snake venoms is due to the oxidative stress induced by hydrogen peroxide in infected cells, resulting in proteolytic activity in treated cells. Subsequently, the mitochondrial function of these cells is compromised due to calcium influx, activating other enzymes such as nitric oxide synthetase and phospholipases. These events culminate in an increased production of free radicals and consequent destruction of genetic material, with the cell entering apoptosis [116].

An LAAO isolated from* B. moojeni* venom presented leishmanicidal activity against promastigote forms of* Leishmania amazonensis* five times higher than that of the crude venom [66, 113]. However, this enzyme showed no activity when tested against amastigote forms, suggesting that this parasite possesses an effective protection system against free radicals and hydrogen peroxide. Pessatti et al. [117] discussed the possibility that while the promastigote is totally deficient in catalase and glutathione peroxidase, the amastigote accumulates high concentrations of the antioxidant enzymes catalase and superoxide dismutase, which were susceptible to the action of the secondary metabolite formed during the oxidation of L-amino acids, justifying effective leishmanicidal activity of this class of toxins.

Murray [118] showed that amastigote forms of* Leishmania donovani* contain three times more catalase and 14 times more glutathione peroxidase than promastigote forms and are therefore four times more resistant to the enzymatic production of hydrogen peroxide, which raised several studies.

Various researchers have attributed the antiprotozoan potential of LAAOs to hydrogen peroxide since the effect disappeared when the enzyme was incubated with catalase [24, 27, 31, 32, 66, 78, 95]. A similar explanation was provided for the trypanocidal effect of LAAOs [27, 66, 95]. svLAAOs may be involved in several other pharmacological activities.

### 9.3. Bactericidal Effect of L-Amino Acid Oxidases

Since Skarnes [119] observed, for the first time, the bactericidal potential of an LAAO isolated from* Crotalus adamanteus* venom, many other snake venom LAAOs have been shown to be effective against bacteria, irrespective of the genus from which they were isolated:* Trimeresurus jerdonii* [70],* Trimeresurus mucrosquamatus* [69],* Bothrops alternatus* [35],* A. halys* [86],* Crotalus durissus cascavella* [31],* Vipera lebetina* [30],* Bothrops pirajai* [32],* Bothrops moojeni* [66],* Naja naja oxiana* [28],* Bothrops pauloensis* [24],* Bothrops jararaca* [78],* Daboia russelii siamensis* [47],* Agkistrodon blomhoffii ussurensis* [19],* Bothrops marajoensis* [20],* Ophiophagus hannah* [120],* Calloselasma rhodostoma* and* Ophiophagus hannah* [18], and* Crotalus durissus cumanensis* [12].

The true bactericidal mode of action of LAAOs is still not completely understood but seems to be related to the oxidized form of the cofactor of the enzyme (FAD or FMN). This cofactor interacts with L-amino acids that can then act on nucleic acids, proteins, and the plasma membrane. Thus, when in contact with the bacterial membrane hydrogen peroxide can provoke lipoperoxidation [13, 31, 49], DNA fragmentation [29, 32, 75], and consequent cell death. The probable mechanisms induced by LAAOs on proteins involve the enzymatic oxidation of L-amino acids [36].

According to Zhang et al. [86] inhibitory activity of AHP-LAAO isolated from* A. h. pallas* venom on the growth of* Bacillus subtilis* (Gram-positive) and* E. coli* (Gram-negative) was observed. When treated with L-vinylglycine, a substrate that reversibly inhibits the oxidative activity of LAAO [121], the enzyme completely lost its bactericidal activity. The same was observed in the presence of catalase. The authors suggested that the bactericidal activity of AHP-LAAO is related to its oxidative potential. Since L-vinylglycine is a substrate that interacts with the catalytic site of LAAOs and not with the glycan moiety, the loss of bactericidal activity in the presence of this substrate indicates that the presence of carbohydrates is not fundamental for this activity.

The LAAO isolated from* Crotalus durissus cascavella* venom by Toyama et al. [31], called CascaLAO, presented bactericidal activity against* Xanthomonas axonopodis pv. passiflorae* (Gram-negative) and* Streptococcus mutans* (Gram-positive). This activity was demonstrated using transmission electron microscopy by rupturing the plasma membrane of microorganisms and observing consequent leakage of cytoplasmic content and cell death. An immunohistochemical study also demonstrated that achacin, an LAAO purified from* Achatina fulica* [26], binds to the plasma membrane of* Staphylococcus aureus* and* E. coli* during the growth phase, causing death of the bacteria by the mechanisms mentioned above.

According to several investigators [31, 76, 86, 122], the most likely mode of action involved in the bactericidal activity of LAAOs is that hydrogen peroxide causes oxidative stress in the target cell, triggering disorganization of the plasma membrane and cytoplasm and consequent cell death.

### 9.4. Cytotoxic and Apoptotic Effects of L-Amino Acid Oxidases

The mechanisms of cytotoxicity induced by LAAOs involve two processes: necrosis and apoptosis. Necrosis is related to the direct action of the enzyme or its catabolic products on the plasma membrane of the cell, promoting its degeneration [29, 36, 123]. In contrast, apoptosis is initiated through various pathways; one of them is the generation of reactive oxygen species and free radicals [124]. One common pathway of cell death involves the products of tumor suppressor genes, including p53, which induce apoptosis. Protein p53 participates in growth inhibition and DNA repair or apoptosis after DNA damage induced by cytotoxic agents [125]. Due to the central role of p53 in many human cancers, including gliomas, its regulation and expression might be a potential target in glial cell cancer therapy [126].

The induction of apoptosis in tumor cells is one of the most important mechanisms of anticancer agents. Apoptotic events coincide with morphological, biochemical, and molecular alterations that lead to cell death. The most frequent morphological alterations include chromatin condensation, disintegration of the nucleolus, and a reduction in cell volume. Biochemical alterations culminate in the production of oxidant enzymes, and molecular changes are associated with the fragmentation of DNA, as shown in Table 5.

According to Ponnudurai et al. [53], this biological effect may result from a secondary action of hydrogen peroxide produced during the oxidation reaction of the substrate. This theory is supported by the finding that the addition of catalase or GSH (glutathione) to LAAOs coincides with the loss of activity of these enzymes. Increasing evidence supporting this hypothesis includes the finding that hydrogen peroxide is a mediator of apoptosis that directly acts on oxidative cell metabolism [4].

Mosmann [127] observed that hydrogen peroxide induces the upregulation of Fas in human endothelial cells and that the activation of tyrosine kinase might be involved in the hydrogen peroxide-induced expression of Fas. Fas is a type I membrane protein belonging to the tumor necrosis factor and nerve growth factor receptor family and mediates a death signal. Thus, Fas-mediated apoptosis in human endothelial cells may contribute to the mechanism of hydrogen peroxide-induced endothelial cell injury.

Several of the LAAOs isolated have been considered cytotoxic, including APIT to Jurkat T cells [34],* Vipera berus berus* LAAO to HeLa and K562 cells [33], BpirLAAO-I to S180 cells and macrophages [32], ABU-LAO to human monocytes and T cells [29], BmooLAAO-I to Ehrlich ascites tumor cells [66], BatroxLAAO to HL-60, Jurkat, B16F10, and PC12 cells [25], ACTX-8 to HeLa cells [29], BjarLAAO-I to Ehrlich ascites tumour [27], BF-LAAO to A549 cells [55], BpLAAO to SKBR3 breast carcinoma and Jurkat leukemia cells [24], BmarLAAO to macrophages [20], Bl-LAAO to LL-24, RKO, HUTU, and MKN-45 cells [17], and LmLAAO to AGS cells-gastric adenocarcinoma and MCF-7 cells-breast adenocarcinoma [15].

DiPietrantonio et al. [128] detected an increased activity of caspase 3 in HL-60 cells exposed to hydrogen peroxide. Caspases are proteases of the cysteine family, which are common signaling molecules of apoptosis. Zhang et al. [129] demonstrated that TSV-LAO isolated from* T. stejnegeri* venom presents cytotoxicity in a human leukemia T cell line (C8166) by inducing chromatin condensation and nuclear morphological changes, which are typical phenomena of apoptosis.

Parallel to the treatment of svLAAOs cells infected with parasites, some authors do a first screening with different concentrations of the toxin on normal cells of the same lineage. The dose range that results in cell viability analyzed by MTT above 80% was adopted as criteria for further experiments. Under these conditions, the cells remain viable, but when they are infected with parasites and treated with different doses of the toxin in the range of previously found good viability, the intracellular multiplication of the parasite is inhibited [32].

## 10. Pharmacological Applications of L-Amino Acid Oxidases

According to Kitani et al. [130], SV-LAAOs have a large potential as cytotoxic drugs, based on cell viability obtained by MTT, according to the protocol described by Mosmann [127]. Previously published data have demonstrated cytotoxic activity to be an important characteristic of these enzymes, which started to receive attention for the development of new antimicrobial agents.

Sun et al. [19] demonstrated a protective effect of rat milk LAAOs on mammary glands. This effect is due to a probable antiseptic action of the enzyme when incorporated into milk. Inflammation and infections in the mammary glands are reduced by the presence of the antimicrobial agent, when continually secreted with milk.

According to Nuutinen and Timonen [6], the basidiomycetes* Laccaria bicolor* and* Hebeloma* spp., which live in symbiosis with tree roots of temperate and boreal forests, express the LAAO enzyme, whose function is to oxidize classical amino acids, generating nitrogen that undergoes mineralization to ammonia (NH^4+^). Later, the nitrogen from this ammonia is utilized in the construction of amino acids by the host plant, besides ensuring the recycling of nitrogen derived from amino acids.

Achacin, an LAAO secreted in the mucus of the giant African snail (*Achatina fulica*), seems to protect this snail against aggressors, with the mucus forming a protective barrier against bacteria and fungi. Another LAAO, called SSAP, which is synthesized by the skin of the rockfish (*Sebastes schlegeli*) in the form of mucus, also exerts protective effects similar to those promoted by achacin [128, 130]. This antimicrobial activity is probably related to the mechanism of action of the protein, which exerts a bactericidal effect by producing hydrogen peroxide and can be used as a natural repellent.

There are few leishmanicidal agents for the current leishmaniasis clinical therapy; in addition, visceral leishmaniasis may affect the liver and spleen and can become potentially lethal. Again, hydrogen peroxide was found to play a key role in the cytotoxic effect of the enzyme. However, partial retention of enzymatic activity after the addition of catalase showed the existence of other unknown mechanisms involved in the leishmanicidal and bactericidal effects of LAAO.

There are few effective drugs for the treatment of leishmaniasis, and in the visceral form this disease can affect the liver and spleen and can become potentially lethal; thus, LAAOs could be used as a potentiator of leishmanicidal action. However, its role as a toxin prevents everyday use, due to its nonspecific action. One way to use these toxins as possible therapeutic drugs is to alleviate them without losing the potential toxic effects, permitting their use in therapies or as models for the development of new drugs.

In 1997, the Korean group of Ahn et al. [108] published a study on the* Ophiophagus hannah* LAAO in which its cytotoxicity to tumors cells was evaluated using radioactively labeled thymidine uptake assays. Cytotoxicity was observed in stomach cancer, murine melanoma (B16F10), fibrosarcoma, colorectal cancer, and Chinese hamster ovary cell lines. Markland [131] suggested that this enzyme probably prevents the adhesion of tumor cells and metastases in the host by inhibiting platelet aggregation, in addition to promoting the attack of natural phagocytic cells of the immune system.

Another activity of svLAAOs is their antifung effect. According to Costa Torres et al. [20], a toxin called BmarLAAO isolated from the snake venom of* Bothrops marajoensis* is able to inhibit the growth of* Candida albicans*. Recently Cheng et al. [132] published a study showing that LAAOs induce apoptosis by DNA fragmentation of* Botrytis cinerea*.

## 11. Concluding Remarks

SvLAAOs, potentially toxic proteins, are present in different genera and families of snakes and are responsible for several biological activities. They catalyze a redox reaction of different groups of amino acids, generating hydrogen peroxide as a catabolic product. This reactive oxygen species so far seems to be the molecule responsible for the pharmacological effects of this class of enzymes.

Different LAAOs have been found to be valuable molecules with possible future applications to the treatment of many diseases and as models for the development of antiviral, antitumor, antiparasitic, and antimicrobial drugs. However, the development of therapeutic agents based on the structure of widely characterized molecules previously isolated from snake venoms is gaining popularity in the search for future drugs.

## Figures and Tables

**Table 1:** Biochemical profile of L-amino acid oxidase isolated from snake venoms.

Toxin	Venom	MW	pI	Specific activity	Purification column	% of venom	Reference
**CdcLAAO**	*Crotalus durissus cumanensis *	55 kDa	8.0	L	Sephacryl S-200, RP-HPLC	ND	[12]
**Cr-LAAO**	*Calloselasma rhodostoma *	ND	ND	ND	ND	ND	[13]
**Oh-LAAO**	*Ophiophagus hannah *	64 kDa*	**ND**	M, L, H, K, I	Sephadex G-100, Q Column, HiTrap Heparin HP	**ND**	[14]
**Lm-LAAO**	*Lachesis muta *	60 kDa	6.28	L	Sephacryl S100, MonoQ	ND	[15]
**DrLAO**	*Daboia russelii *	63.6 kDa	**ND**	L	Superdex 75, Mono Q, Heparin-Sepharose	0.9%	[16]
**Bl-LAAO**	*Bothrops leucurus *	57 kDa	**ND**	L	Sephacryl S-200, Sephacryl S-300, DEAE Sepharose CL-6B	3.7%	[17]
**ND**	*Ophiophagus hannah *	65 kDa	**ND**	ND	Resource Q	ND	[18]
**Akbu-LAAO**	*Agkistrodon blomhoffii ussuriensis *	65 kDa	**ND**	L	DEAE Sephadex A-50 ion-exchange, Sephadex G-75 gel filtration and C4 reverse phase	5%	[19]
**BmarLAAO**	*Bothrops marajoensis *	72 kDa	**ND**	**ND**	Protein Pack SP 5PW HPLC, Protein Pack SP 5PW anion exchanger, Superdex 200	**ND**	[20]
**LAO *B. caeruleus***	*Bungarus caeruleus *	55 kDa	**ND**	E, L, M, I, F, R	DEAE Cellulose, Sephadex G-100	25%	[21]
**BF-LAAO**	*Bungarus fasciatus *	55 kDa	ND	Y, D, F, E, W, H, Q, I, M, L	SP-Sepharose HP, Heparin-Sepharose FF	0.93%	[22]
***Bothrops jararaca* LAAO**	*Bothrops jararaca *	38.2 kDa*	**ND**	F, Y, L, I	MonoQ, Heparin	1.1%	[23]
**Bp-LAAO**	*Bothrops pauloensis *	65 kDa*	6.3	M, L, F,I	CM-Sepharose, Phenyl-Sepharose CL-4B, Benzamidine Sepharose and C18 RP-HPLC	**ND**	[24]
**BatroxLAAO**	*Bothrops atrox *	67 kDa	4.4	M, L, F, W, Y, I	G-75, HPLC-Shodex ES-502N 7C, Lentil Lectin	1.54%	[25]
**BiLAO**	*Bothrops insularis *	68 kDa	**ND**	**ND**	HPLC AP1, Superdex 75	**ND**	[26]
**BjarLAAO-I**	*Bothrops jararaca *	60 kDa	5.0	**ND**	Sephadex G-75, Benzamidine-Sepharose, Phenyl-Sepharose	**ND**	[27]
***N. naja oxiana* L-amino acid oxidase**	*Naja naja oxiana *	57 kDa	8.0	M, L, F, W	Sephadex G-50 SF, CM-cellulose CM52, HPS-7	0.15%	[28]
**ACTX-8**	*Agkistrodon acutus *	28 kDa	8.2	**ND**	DEAE Sepharose F. F., Source 30	**ND**	[29]
**Akbu-LAAO**	*Agkistrodon blomhoffii ussurensis *	58–60 kDa	**ND**	N, F, Y, L, I, W	Heparin-Sepharose FF, (Q-Sepharose)	1.27%	[19]
***Vipera lebetina* LAAO**	*Vipera lebetina *	66 kDa** or 60.9 kDa*	4.5	M, W, L, H, F, R, I	Sephadex G-100, HPS-7, DEAE-cellulose DE52., CM-cellulose CM52	2.5%.	[30]
**Casca-LAO**	*Crotalus durissus cascavella *	68 kDa	5.4	ND	Superdex 75	0.28%	[31]
**BpirLAAOI**	*Bothrops pirajai *	66 kDa	4.9	F, Y, W, L, M, I, V, H	Sephadex G-75, Benzamidine-Sepharose, Phenyl-Sepharose.	**ND**	[32]
***V. berus berus* LAAO**	*Vipera berus berus *	59 kDa, or 57.7** kDa	4.8	M, L, F, I, R, H	Sephadex G-100, DEAE-cellulose, phenyl-agarose	1.8%	[33]
**APIT**	*Aplysia punctata *	60 kDa	4.59	K, R	Source 15Q 10/40, Superose 12 HR 10/30	**ND**	[34]
**Balt-LAAO-I**	*Bothrops alternatus *	66 kDa	5.37	F, Y, M, L	Sepharose-IDA, Phenyl-Sepharose, Sephadex G-100	1.0%	[35]

*Deglycosylated protein. **Estimated by MALDI-TOF. ND: not determined.

**Table 2:** Kinetic parameters of L-amino acid oxidase from snake venom on specific substrates.

Snake	Leu		Met		Trp		Phe		Reference
	*K*_*m*_ (mM)	*K*_cat_ (s^−1^)	*K*_*m*_ (mM)	*K*_cat_ (s^−1^)	*K*_*m*_ (mM)	*K*_cat_ (s^−1^)	*K*_*m*_ (mM)	*K*_cat_ (s^−1^)	
*Crotalus durissus cumanensis *	9.23	1.8	ND	ND	ND	ND	ND	ND	[12]
*Daboia russelii *	490	2.153	373	2.193	81	4.056	142	4.130	[16]
*Lachesis muta *	0.97	ND	ND	ND	ND	ND	ND	ND	[15]
*Bungarus fasciatus *	60.69	1025.05	15.03	589.33	0.27	98.82	84.08	142.62	[55]
*Naja naja oxiana *	0.75	47.98	0.885	66.26	0.147	18.04	0.051	17.18	[28]
*Agkistrodon blomhoffii ussurensis *	0.11	48.22	0.88	24.13	0.023	6.58	0.042	48.23	[19]
*Vipera berus berus *	0.361	75.16	0.286	74.20	—	—	0.058	28.50	[33]
*Vipera lebetina *	0.40	52.0	0.65	80.3	0.17	42.65	—	—	[30]
*Naja naja kaouthia *	0.66	23.4	0.63	24.4	0.29	12.47	0.06	10.75	[56]
*Calloselasma rhodostoma *	0.63	3.30	0.24	1.65	0.08	0.88	0.05	0.72	[53]
*Ophiophagus hannah *	0.20	96.2	0.63	65.6	0.10	32.1	0.10	54.1	[57]

ND: not determined.

**Table 3:** Sequence of snake venom L-amino acid oxidase deposited in the NCBI database.

Family	Snake	bp*	gi	Reference
Viperidae	*B. n. pauloensis *	1519	195927837	[24]
Viperidae	*Bothrops jararaca *	1452	ND	[27]
Viperidae	*Viridovipera stejnegeri *	1551	33355626	[111]
Viperidae	*Bitis gabonica *	180	38000585	[22]
Viperidae	*Bothrops moojeni *	1436	398441345	[95]
Viperidae	*Bothrops jararacussu *	1491	398441343	[95]
Viperidae	*Crotalus adamanteus *	2787	3426323	[94]
Elapidae	*Bungarus fasciatus *	2815	126035652	[14]
Elapidae	*Naja atra *	1347	126035676	[14]
Elapidae	*Bungarus multicinctus *	2794	126035648	[14]
Elapidae	*Ophiophagus hannah *	2883	126035643	[14]

ND: not determined. *bp: base pairs.

**Table 4:** Percent of similarity between L-amino acid oxidases from snake venoms.

Sequences	GI	(1)	(2)	(3)	(4)	(5)	(6)	(7)	(8)	(9)
*Calloselasma rhodostoma *	20141785	*100.00 *	85.36	85.99	88.27	88.76	89.48	86.63	84.69	88.32
*Bothrops moojeni *	82127389	85.36	*100.00 *	87.39	88.77	88.70	88.49	86.40	95.82	88.08
*Crotalus adamanteus *	6093636	85.99	87.39	*100.00 *	87.19	87.94	87.65	85.41	87.35	87.67
*Agkistrodon halys *	48425312	88.27	88.77	87.19	*100.00 *	91.77	99.18	90.95	90.12	90.74
*Ovophis okinavensis *	538260091	88.76	88.70	87.94	91.77	*100.00 *	92.66	89.53	88.95	92.67
*Gloydius blomhoffii *	75570145	89.48	88.49	87.65	99.18	92.66	*100.00 *	91.67	91.07	91.47
*Trimeresurus stejnegeri *	33355627	86.63	86.40	85.41	90.95	89.53	91.67	*100.00 *	86.24	91.49
*Bothropoides pauloensis *	347602324	84.69	95.82	87.35	90.12	88.95	91.07	86.24	*100.00 *	91.09
*Protobothrops flavoviridis *	538259837	88.32	88.08	87.67	90.74	92.67	91.47	91.49	91.09	*100.00 *

**Table 5:** Cytotoxic and apoptotic effects of L-amino acid oxidases isolated from snake venom.

Venom	Toxin	Cell line (DNA)	Concentration and treatment time	Reference
*Lachesis muta *	LmLAAO	LL-24, AGS, MCF-7, and HUTU	1.17–75 *μ*g/mL for 24 h	[15]
*Bothrops leucurus *	Bl-LAAO	MKN-45 and RKO	0.1–20 *μ*g/mL for 24 h	[17]
*Bungarus fasciatus *	BF-LAAO	A549	0.03–3.0 *μ*g/mL for 12 h	[55]
*Bothrops atrox *	LAAO	PC12, B16F10, HL-60, and Jurkat	5–50 *μ*g/mL for 4 h	[45]
*Bothrops moojeni *	BmooLAAO-I	HL-60	8–16 *μ*g/mL for 12 h	[66]
*Agkistrodon acutus *	ACTX-8	HeLa	20 *μ*g/mL for 12–48 h	[29]
*Bothrops pirajai *	BpirLAAO-I	Fago M13mp18	1–20 *μ*g/mL for 24 h	[32]
*Vipera berus berus *	*V. berus berus* LAAO	HeLa and K562	2.5–10 *μ*g/mL for 7–24 h	[33]
*Trimeresurus flavoviridis *	OHAP-1	RBR17T and C6	2.5 and 5 *μ*g/mL for 24 h	[48]
*Eristicophis macmahoni *	LNV-LAO	MM6	25–100 *μ*g/mL for 18 h	[83]
*Agkistrodon contortrix laticinctus *	ACL LAO	HL-60	2.5–100 *μ*g/mL for 16 h	[75]
